# PTEN as a Prognostic and Predictive Marker in Postoperative Radiotherapy for Squamous Cell Cancer of the Head and Neck

**DOI:** 10.1371/journal.pone.0033396

**Published:** 2012-03-07

**Authors:** Miroslaw Snietura, Magdalena Jaworska, Joanna Mlynarczyk-Liszka, Aleksandra Goraj-Zajac, Wojciech Piglowski, Dariusz Lange, Grzegorz Wozniak, Elzbieta Nowara, Rafal Suwinski

**Affiliations:** 1 Tumor Pathology Department, Maria Sklodowska-Curie Memorial Cancer Center and Institute of Oncology, Gliwice Branch, Poland; 2 Department of Radiation Oncology, Maria Sklodowska-Curie Memorial Cancer Center and Institute of Oncology, Gliwice Branch, Poland; 3 Department of Clinical and Experimental Oncology, Maria Sklodowska-Curie Memorial Cancer Center and Institute of Oncology, Gliwice Branch, Poland; Karolinska Institutet, Sweden

## Abstract

**Background:**

Tumor suppressor PTEN is known to control a variety of processes related to cell survival, proliferation, and growth. PTEN expression is considered as a prognostic factor in some human neoplasms like breast, prostate, and thyroid cancer.

**Methodology/Principal Findings:**

In this study we analyzed the influence of PTEN expression on the outcome of a randomized clinical trial of conventional versus 7-days-a-week postoperative radiotherapy for squamous cell cancer of the head and neck. The patients with cancer of the oral cavity, oropharynx, and larynx were randomized to receive 63 Gy in fractions of 1.8 Gy given 5 days a week (CF) or 7 days a week (p-CAIR). Out of 279 patients enrolled in the study, 147 paraffin blocks were available for an immunohistochemical assessment of PTEN. To evaluate the prognostic value of PTEN expression and the effect of fractionation relative to PTEN, the data on the outcome of a randomized clinical trial were analyzed. Tumors with a high intensity of PTEN staining had significant gain in the loco-regional control (LRC) from p-CAIR (5-year LRC 92.7% vs. 70.8%, for p-CAIR vs. CF, p = 0.016, RR = 0.26). By contrast, tumors with low intensity of PTEN did not gain from p-CAIR (5-year LRC 56.2% vs. 47.2%, p = 0.49, RR = 0.94). The intensity of PTEN highly affected the LRC in a whole group of 147 patients (5-year LRC 80.9% vs. 52.3% for high vs. low PTEN, p = 0.0007, RR = 0.32). In multivariate Cox analysis, including neck node involvement, EGFR, nm23, Ki-67, p53, cyclin D1, tumor site and margins, PTEN remained an independent predictor of LRC (RR = 2.8 p = 0.004).

**Conclusions/Significance:**

These results suggest that PTEN may serve as a potent prognostic and predictive marker in postoperative radiotherapy for high-risk squamous cell cancer of the head and neck.

## Introduction

Tumor suppressor PTEN (Phosphatase and tensin homolog deleted on chromosome 10) is a dual-specific phosphatase that acts as a negative regulator of the PI3K-AKT-mTOR pathway, thus controlling a variety of processes related to cell survival, proliferation, and growth. PTEN performs a crucial role in the silencing of signal transduction from membrane growth factor receptors (EGFR, HER-2, IGFR) through the AKT signaling cascade [1], [2].

Somatic PTEN mutations and deletions or epigenetic silencing are common in multiple tumor types, including breast, endometrium, and thyroid, but also tumors of central nervous system, prostate, lung, melanoma, leukemia and lymphoma [3]–[11]. Loss of PTEN can lead to tissue-specific effects, including rapid or slow tumors, or no tumors. In many neoplasms PTEN deletion cooperates with other genetic alternations to enhance tumorigenesis and may determine aggressive clinical behavior of the tumor (e.g. anaplastic thyroid carcinomas or grade IV invasive astrocytomas) [12].

Because the AKT pathway dictates multiple downstream processes, including inhibition of apoptosis, tumor-cell proliferation [13], and DNA repair [14], and is also known to be associated with radioresistance mechanisms, such as intrinsic radioresistance and hypoxia [15], inactivation of PTEN may affect the effectiveness of anticancer therapy.

These factors form the basis of an increased interest in PTEN and other elements of the AKT pathway as the potential prognostic and predictive markers in combined modality treatment, as well as the targets for new drugs [16].

While the molecular functions of PTEN within the cell are relatively well established, there are only a few clinical studies that would address its prognostic and predictive value in radiotherapy for cancer of the head and neck. We have recently published results of a study on molecular predictors of the effect of accelerated (7 days a week) postoperative radiotherapy. It was based on the analysis of loco-regional control rates obtained in a p-CAIR (postoperative continuous accelerated irradiation) trial in head and neck [17] relative to the expression of five molecular markers (EGFR, Ki-67, p-53, nm23, and cyclin D) [18] and the presence of HPV infection [19].

## Methods

### Objectives

The purpose of the present study is to evaluate the role of PTEN as a prognostic and predictive factor in postoperative radiotherapy for cancer of the head and neck. In addition, we also examine the relationship between the expression of PTEN and markers previously studied in this cancer type.

### Clinical background of the study

The p-CAIR trial was performed between 2001 and 2004, and recruited 279 patients with high-risk squamous cell cancer of the larynx (158 patients) or cancer of the oral cavity/oropharynx (121 patients). Clinical characteristics of the whole group of 279 patients, a detailed presentation of the risk assessment scale, treatment description, and the clinical outcome have been presented elsewhere [17]. The patients were randomized to receive 63 Gy in fractions of 1.8 Gy, given either 5 days a week (140 patients: p-CF, postoperative conventional fractionation) or 7 days a week (139 patients: p-CAIR).

Recently, we presented analysis of the immunohistochemically assessed expression of five molecular markers (EGFR, nm23, Ki-67, p53 and cyclin D1) [18] and Real-Time PCR-assessed presence of HPV infection [19] in relation to the outcome of the trial. For the purpose of PTEN assessment, it was possible to obtain material from 147 out of 279 patients who were originally enrolled. The follow-up data have been updated in the present study and show the status of the patients as of January 2010 (median follow-up time: 87 months).

We note that the update does not affect the qualitative outcome of the analysis, because new loco-regional recurrences have not been recorded since the last publication.

### Participants

Table 1 presents clinical characteristics of 147 patients included in the analysis. The general characteristics of the subgroup analyzed in the present study did not differ considerably from that of the population of 279 patients [17].

**Table 1:** Characteristics of 147 patients with high-risk squamous cell cancer of the head and neck.

	p-CF (N = 73)	p-CAIR (N = 74)	p-value
Age (median, range)	59 (39–71)	57 (41–77)	0.61
Gender	F: 6 (8.2%)M: 67 (91.8%)	F: 9 (12.2%)M: 65 (87.8%)	0.17
T	T1,T2: 20 (27.8%)T3,T4: 53 (72.2%)	T1,T2: 17 (22.9%)T3,T4: 57 (77.1%)	0.54
N	N0: 26 (36.1%)N+: 47 (63.9%)	N0: 18 (24.3%)N+: 37 (75.7%)	0.73
Tumor site	Oral cavity: 28 (38.4%)Oropharynx: 9 (12.3%)Hypopharynx/larynx: 36 (49.3%)	Oral cavity: 23 (31.1%)Oropharynx: 12 (16.2%)Hypopharynx/Larynx: 39 (52.7%)	0.81
Margins	Neg.: 36 (49.3%)Pos.: 26 (35.6%)Uncertain: 11 (15.1%)	Neg.: 47 (63.5%)Pos.: 23 (31.1%)Uncertain: 4 (5.4%)	0.28
Grade	G1: 16 (21.9%)G2: 42 (57.5%)G3: 15 (20.6%)	G1: 10 (13.5%)G2: 45 (60.8%)G3: 19 (25.7%)	0.08
HPV status	Pos.: 5 (6.9%)Neg.: 60 (82.2%)Uncertain: 8 (10.9%)	Pos.: 4 (5.4%)Neg.: 62 (83.8%)Uncertain: 8 (10.8%)	0.71

p-CF: postoperative conventional fractionation.

p-CAIR: postoperative continuous accelerated fractionation.

N+: lymph node involvement.

The majority of individuals participating in both arms of the study were males (∼90%). Such gender prevalence is representative for a population of HNSCC in Poland and is associated with smoking habits and alcohol consumption.

Importantly, the risk factors for recurrence were evenly distributed between the trial arms. The actual mean overall radiation treatment time among 131 patients was 48.4 days (SD = 6.3) in CF vs. 35.6 (SD = 6.8) in p-CAIR. Four patients in p-CF and four in p-CAIR received total radiation doses lower than planned, due to deteriorating general performance before or during radiotherapy. Four patients in p-CF and three in p-CAIR received total doses higher than in the protocol, due to local/nodal tumor progression during treatment or treatment planning. One patient in the p-CAIR arm died before radiotherapy was started. The other 131 patients received 63 Gy in 1.8 Gy fractions. None of the patients received chemotherapy. The analysis, as shown, was performed on an intention-to-treat basis.

### The analysis of PTEN expression

The PTEN expression pattern was examined immunohistochemically in formalin-fixed, paraffin-embedded tumor samples from all patients enrolled.

The monoclonal, rabbit, anti-human PTEN antibody, clone 138G6, raised against the last 100 C-terminal aminoacids of the protein, was used in all analyses (Cell Signaling Technology, Denver). The PTEN specificity of this antibody was confirmed using Western blotting and immunohistochemistry techniques in several cell lines with known PTEN status. Immunostaining was performed according to standard guidelines: four-micrometer sections were cut, mounted on slides, deparaffinized, and rehydrated in xylene and in a graded series of ethanol. Epitope retrieval was performed for 20 minutes at 98°C in TRIS buffer/EDTA 10 mmol/l, pH 9.0. Endogenous peroxidase activity was inactivated by 3% solution of H_2_O_2_ for 5 min. Next, primary antibody was applied to the slides (1∶100 dilution in Background Reducing Antibody Diluent, Dako, Denmark) and incubated overnight at 4°C. Finally, a mixture of polymer conjugated with horseradish peroxidase and affinity-purified goat anti-rabbit Fab' antibody fragments and DAB chromogen was used to visualize the reaction (EnVision+ Dako, Denmark). After counterstaining in hematoxylin, the specimens were covered with AquaMount (Dako, Denmark) mounting medium.

**Figure 1:**
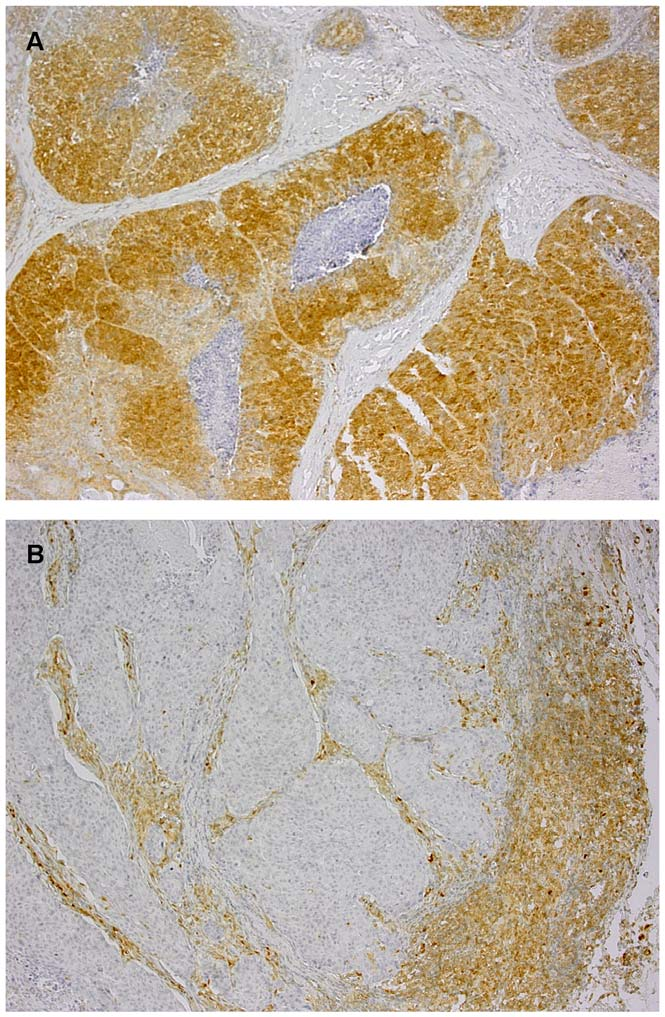
An expression of PTEN in the tumor tissue. A range of PTEN expression in the tumor tissue: A - high, B - low. Visible positive staining of the surrounding matrix in both specimens. Original magnification: 100×.

Staining intensity was evaluated by a pathologist, who was blinded to the clinical information pertaining to patients. Scoring was performed according to a relative semi-quantification method described originally by Perren [20] and widely used in many recent studies concerning head and neck tumors [21], melanoma [22], breast [3], [23], ovarian [24], and colorectal [25] cancers. Representative areas of tumor were selected and scored under original magnification ×200. Slides were classified into two groups, depending on the staining intensity of tumor cells in relation to PTEN expression in adjacent normal-appearing epithelium or stroma, which served also as an internal, positive control. Tumors showing no staining or with staining intensity lower than in corresponding normal tissue were classified as LOW PTEN (Fig. 1B), while those with increased or equal expression were considered to be HIGH PTEN (Fig. 1A).

A series of histological preparations with known PTEN status served as positive control. Additional tumor sections, treated as described before but without primary antibody application, revealed no staining (negative control).

### Ethics

The Ethical Committee of the Maria Sklodowska-Curie Memorial Cancer Center and Institute of Oncology, Gliwice Branch specifically approved this study according to the national regulations. A written, informed consent was obtained for all participants of the study.

### Statistical methods

Loco-regional failure was defined as the recurrence of cancer at the primary tumor site, within the neck or supraclavicular nodes, and distant metastases as the recurrence elsewhere. The survival curves have been plotted using the Kaplan-Meier method, and compared using the Cox f test. Taking into account the exploratory purpose of this research and the relatively small number of patients, the Cox f test was considered to be more suitable for testing the differences between two groups, than was the more commonly used log-rank test, designed for use in larger studies. To further explore the prognostic significance of variables of importance, a univariate and multivariate Cox proportional hazard regression analysis was performed. Only the variables that appeared statistically significant in univariate analysis were included in the multivariate model. The model was optimized using a backward stepwise regression.

## Results

### The prognostic value of PTEN expression

Low PTEN expression was detected in 88/147 tumors (59.9%). Among 74 patients with laryngeal cancer, 46 (62.2%) had low expression of PTEN compared to 42/73 (57.3%) patients with cancer of the oral cavity/oropharynx. Neither T stage (p = 0.10), nor N stage at the diagnosis (p = 0.86) were related to PTEN expression, as evaluated by the U-Mann Whitney test. PTEN expression was, however, significantly affected by gender of the patients. Low PTEN expression was detected in 4/15 (26.7%) females, compared to 84/132 (63.6%) males (p = 0.006, U-Mann Whitney test).

**Figure 2:**
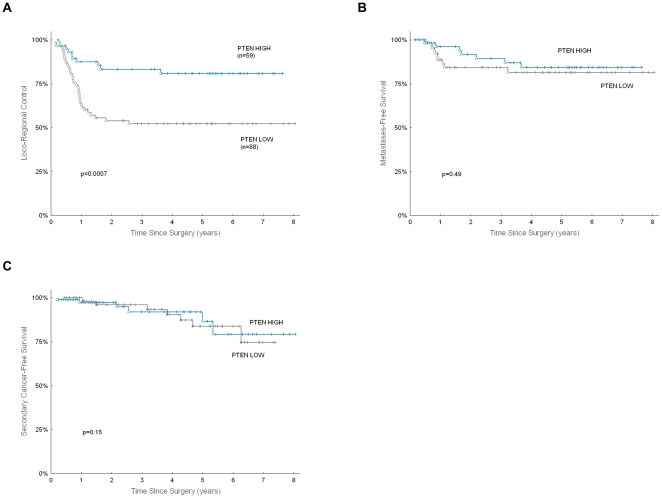
Clinical outcome according to PTEN expression. Loco-regional control (A), distant metastases-free survival (B), and freedom from secondary cancer (C), according to PTEN expression in 147 patients with high-risk HNSCC treated in p-CAIR trial.

Loco-regional tumor control was highly affected by PTEN expression: 5-year LRC was 52.3% in patients with low PTEN expression, compared to 80.9% in patients with high PTEN expression (p = 0.0007, Fig. 2A). The prognostic significance of PTEN was apparent both for local control (5-year LC 63.9% vs. 84.8% for low vs. high PTEN, p = 0.01) and for regional (nodal) control (5-year NC 74.6% vs. 87.9% for low vs. high PTEN, p = 0.05).

Five-year metastases-free survival was 81.4% in patients with low PTEN expression, vs. 84.3% in patients with high PTEN expression; the difference did not appear significant (p = 0.49, Fig. 2B). Also, the accumulated rate of metachronous tumors was not significantly affected by PTEN expression (p = 0.15, Fig. 2C).

### The predictive value of PTEN expression

**Figure 3:**
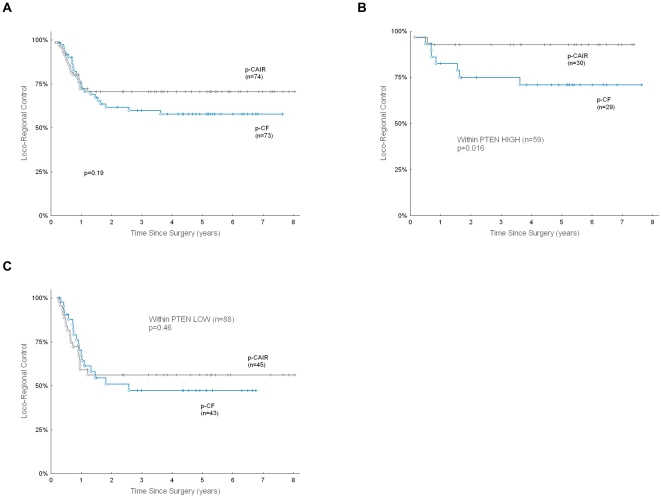
PTEN expression as a predictor of benefits from p-CAIR fractionation. Loco-regional control (LRC), according to fractionation in subset of 147 patients from the p-CAIR trial used in the present study (A), LRC according to fractionation and expression of PTEN in subset of 147 patients from the p-CAIR trial used in the present study: HIGH PTEN expression (B), LOW PTEN expression (C).

Because p-CAIR trial was designed to compare loco-regional tumor control in accelerated vs. conventional postoperative radiotherapy, it was possible to evaluate the predictive potential of PTEN expression relative to fractionation. The clinical outcome in a subset of 147 patients appeared representative for the outcome observed in all 279 patients: 5-year loco-regional tumor control was 57.6% in CF, compared to 70.6% in p-CAIR; the difference was not statistically significant (Fig. 3A, p = 0.19).

In a subgroup of 88 patients with low PTEN expression, 5-year LRC was 47.2% in CF, compared to 56.2% in p-CAIR (Fig. 3C, p = 0.19). In a subgroup of 59 patients with high PTEN expression, 5-year LRC was 70.8% in CF, compared to 92.7% in p-CAIR (Fig. 3B, p = 0.016). Thus, low PTEN expression was related not only to unfavorable LRC, but also to lack of improvement in LRC from accelerated fractionation.

### PTEN versus other variables and markers

The expression of PTEN correlated neither with HPV infection, nor with expression of any of five previously studied markers (EGFR, nm23, Ki-67, P53, cyclin D1), as indicated by p>0.05 in all performed U-Mann Whitney tests.

A multivariate model designed to assess an independent prognostic significance of variables of potential importance for LRC initially incorporated several factors, including expression of PTEN, EGFR, nm23, Ki-67, p-53, and cyclin D1; clinical variables such as age, gender, performance status, tumor site; and classical pathological risk factors, such as neck node involvement, margins, and tumor grade. HPV infection was not incorporated into the model because only nine patients appeared to be HPV-positive in this group, and all were locally controlled (no uncensored data in the HPV-positive group) [19].

After we eliminated non-significant and confounding variables from the model, only four variables appeared to be significantly and independently related to LRC: PTEN expression (RR = 2.84, p = 0.004), neck node involvement (RR = 2.05, p = 0.035), EGFR expression (RR = 1.91, p = 0.033) and nm23 expression (RR = 1.88, p = 0.041, Table 2).

**Table 2:** Factors that significantly and independently affected LRC: results of a multivariate analysis.

Variable	RR	95% CI for RR	p-value
Low PTEN expression	2.84	1.38–5.80	0.004
Neck node involvement	2.05	1.04–4.00	0.035
High EGFR expression	1.91	1.05–3.48	0.033
High nm23 expression	1.88	1.09–3.65	0.041

RR: Relative Risk; CI: confidence interval.

## Discussion

### PTEN as a prognostic marker

Several clinical and pathological factors have been identified to prognose risk of recurrence after surgery for locally advanced HNSCC [26], [27]. These factors are used to select candidates for postoperative radiotherapy and to optimize radiation dose. More recently, based on results of large randomized clinical trials [28], [29], the patients at high risk of recurrence are considered for postoperative radiochemotherapy. The “conventional” risk factors such as positive surgical margins, invasion of more than one neck node, extracapsular spread of nodal disease, and oral cavity/oropharyngeal primary tumor site, except T1N0, were included among selection criteria for a p-CAIR trial [17].

Recent advances in immunochemistry and molecular biology have facilitated the identification of new markers that help to prognose risk of recurrence. At least two of them (HPV infection and EGFR expression) are progressively more recognized in a routine clinical practice [30], [31]. The results of the present study suggest that PTEN might become another marker of major clinical importance.

In our study we went further and tested a multivariate model, including other potential markers (Table 2). PTEN appeared to have a stronger prognostic value than any of the other variables considered, including neck node involvement and EGFR expression. Only one of the variables used in a “conventional” evaluation of risk factors remained significant when confronted with new markers. We could not compare the prognostic value of PTEN and HPV infection in the present study, because lack of uncensored data in HPV-positive group precluded such analysis. Nevertheless, lack of correlation between PTEN expression and HPV infection indicate that these two variables are independent.

The analysis, as shown, refer to our recently published results on the prognostic and predictive value of five molecular markers (EGFR, Ki-67, P53, nm23, and cyclin D) [18] and the presence of HPV infection [19]. We had proposed a prognostic index for recurrence after postoperative radiotherapy that incorporated three factors of the greatest importance: expression of EGFR, expression of nm23, and invasion of the neck nodes [18]. The results of the present analysis and the emerging data on HPV infection [19], [30] prompt us to propose a new, improved score that would incorporate five risk factors of foremost importance for the outcome of postoperative radiotherapy for HNSCC:

- absence of HPV infection in tumor
- low PTEN expression
- neck node involvement
- high EGFR expression
- high nm23 expression

**Figure 4:**
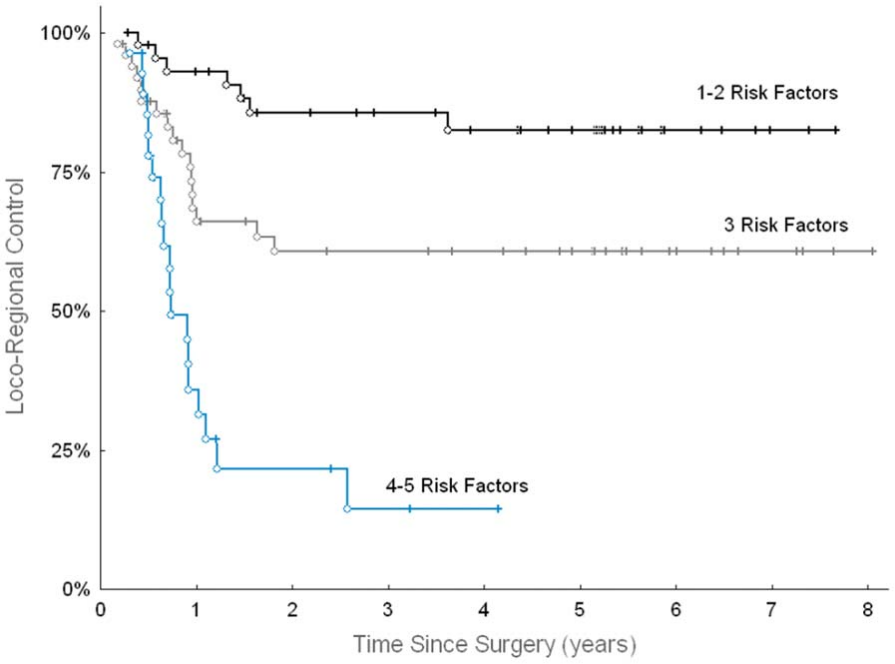
Loco-regional control in HNSCC according to most significant risk factors. Loco-regional control according to the number of following risk factors: absence of HPV infection in tumor, low PTEN expression, neck node involvement, high EGFR expression, and high nm23 expression. The analysis was restricted to 123 patients from the p-CAIR trial with known HPV status and known status of the other 4 markers.

Figure 4 illustrates LRC in the p-CAIR trial, according to the number of such defined risk factors. Because not all of the patients included in the present study had established HPV status, only the data from 123 patients with known status of all five markers were used to plot the graph. None of the patients had zero risk factors in the analyzed group, mostly because the prevalence of HPV infection was low (6.9%) in this group. We expect that ongoing research on molecular markers in cancer performed by other investigators will help to validate and further improve this prognostic score.

### PTEN as a predictive marker

Several clinical studies demonstrated the potential of molecular profiles to predict the benefit from accelerated radiotherapy [31]–[33], including our previous report on molecular markers in the p-CAIR trial [18].

The results of the present study indicate that the expression of PTEN may be useful in predicting the benefit from accelerated radiotherapy: the patients with high PTEN expression have a favourable outcome after accelerated postoperative radiotherapy, compared to the patients treated with conventional fractionation and to those with low PTEN expression (Fig. 3B, C).

The clinical significance of this finding is limited by current evidence-based prescription practice that would favour postoperative radiochemotherapy over postoperative radiotherapy alone in high-risk HNSCC [27]–[29]. However, the emerging data on molecular markers and HPV infection raise several questions over actual need of chemoradiotherapy in patients who would, likely, have favourable outcome after postoperative radiotherapy alone. We hope that future clinical trials and research on molecular markers will further address this important concern.

### The biological background of the present study

A possible role of PTEN loss in the pathogenesis of HNSCC has been described by Califano [34] in his stepwise model of carcinogenesis. Later, a whole spectrum of functional and structural dysfunctions of PTEN in HNSCC was demonstrated, including mutations, losses of heterozygocity (LOH), and epigenetic silencing [35]–[38]. The majority of these disorders lead to a decrease or loss of protein expression, thus giving the rationale for defective PTEN detection by immunohistochemistry.

Recently, a new model of PTEN role in cancer development has been proposed and verified in vivo. According to this model, subtle reduction (20%) in dose of PTEN, altered the biology of cancer and the expression profiles of genes involved in a cancer cell proliferation [39], [40].

Because of the diverse expression of PTEN in normal tissues and individual tumors, as well as the crucial role of subtle variations of PTEN concentration for cancer phenotype development [12], there is a need for a more sophisticated immunohistochemistry scoring method. A strategy, proposed by Perren and colleagues [20], to use normal tissue or stroma as a common reference providing a cut-off point for normal/dysfunctional PTEN, ensures case-to-case comparable results and was implemented in our study. The same methodology was used by Lee and co-workers [21], who investigated PTEN expression in patients with tongue cancer and showed that loss of PTEN is connected with shorter overall survival and event-free survival time. Furthermore, PTEN status remained an independent predictor of poor outcome when compared with tumor stage and nodal status. Similarly, the significance of PTEN expression as a marker of favourable outcome in combined modalities comprising irradiation has been reported for many other cancers, including astrocytomas [41], cervical [4], [42] and breast cancer [20], prostate [7], [8] and colorectal cancer [43].

More ambiguous results have been shown by Tsutsui [23] and Perez-Tonorio [3] in the context of breast cancer. They demonstrated that prognosis more clearly depended on the combined status of PTEN/P53 and PTEN/S-phase fraction, respectively.

By contrast, Pattje and colleagues [44] recently showed that there is an increased risk of locoregional failure after postoperative radiotherapy in patients with head and neck squamous cell cancer expressing PTEN. As a proof of concept, the authors showed a decreased radiosensitivity in a human embryonic kidney cell line (Hek293) with an overexpressed PTEN gene. Despite the superficial similarities to the present studies, including tumor sites, treatment modality or HPV infection frequency, there are many essential differences in the experimental group and design, between our approach and the above-mentioned research. The present study was performed in a prospective clinical trial setting, ensuring strictly defined criteria of patient recruitment, uniformity of doses, and fractionation among the patients. Also, gender of individuals participating in both arms of the trial was heavily skewed toward males (∼90%) and correlated with tobacco smoking, as opposed to the cited paper, where both genders were distributed evenly.

A different methodology of immunohistochemistry scoring is another substantial difference that could contribute to a conflicting result. Pattje and coworkers used a less popular, absolute method of semi-quantification [44], [45], where any positive staining above background was considered as positive. This fact is of crucial importance in light of the new evidence, demonstrating that even a small decrease of PTEN activity can change the biology of the tumor and that complete loss of functionality may lead to the opposite effects, then a subtle variation [40] in its activity. The methodology implemented by Pattje may cause, that only cases with complete loss of the protein expression are classified as PTEN-dysfunctional. In the present study, a relative semi-quantification method was utilized; thus, tumors with decreased PTEN expression in relation to normal tissue were considered as PTEN-dysfunctional. Such an approach seems to be consistent with new concepts of PTEN functioning. It is also worth noting that relative semi-quantification could be difficult to implement in the case of the tissue matrix used in Pattje's study, due to the scant contents of normal tissue in TMA cores.

An interesting concept, namely “PTEN paradigm” or “obligate haplo-insufficiency” introduced recently, may provide an additional explanation of these conflicting results. According to this theory, PTEN cellular functions are tightly bound to P53 status. In the case of complete PTEN loss, functional P53 directs the cell to acute cellular senescence. Thus, heterozygous loss of PTEN is more tumorigenic than complete loss of PTEN in the context of wild-type P53. This pattern changes in the setting of mutated P53 cells that are unable to activate P53 do not undergo PTEN loss-induced cellular senescence [46]. As a result, complete loss of PTEN cooperates synergistically with partial or complete loss of P53 [47]. Most probably, because of the high consumption of tobacco and low proportion of HPV-positive cases (which are mutually exclusive to P53 mutations), a prevalence of P53 mutations in the studied groups of patients was high (75.4%, as confirmed by immunohistochemistry) [18]. In accordance with the PTEN paradigm, synergistic effects could be expected in a case of PTEN/P53-defective patients, leading to the favourable prognosis in PTEN-positive individuals.

In conclusion, the results of the present study and other studies demonstrate that the clinical usefulness of PTEN as the prognostic or predictive marker for radiotherapy is not yet robustly defined. Therefore, there is a clear need for new research that would further address these interesting issues with reference to combined treatment modalities.
